# Multi-source dataset of e-commerce products with attributes for property matching

**DOI:** 10.1016/j.dib.2022.107884

**Published:** 2022-02-02

**Authors:** Daniel Ayala, Inma Hernández, David Ruiz, Erhard Rahm

**Affiliations:** a Universidad de Sevilla, ETSII, Avda. Reina Mercedes, s/n, Sevilla, Spain; b Leipzig University, Institus für Informatik, Leipzig 04109, Germany

**Keywords:** Property matching, Data integration, Ontology, Data engineering

## Abstract

Schema/ontology matching consists in finding matches between types, properties and entities in heterogeneous sources of data in order to integrate them, which has become increasingly relevant with the development of web technologies and open data initiatives. One of the involved tasks is the matching of data properties, which attempts to try to find correspondences between the attributes of the entities. This is challenging due to the at times different names of equivalent properties. Furthermore, some properties may not be equivalent, but still match in 1..n relationships. These difficulties create the need for varied evaluation datasets for two reasons. First, they are needed to evaluate existing techniques in a variety of scenarios. Second, they enable the training of supervised techniques that may even become context-independent if trained with data from diverse enough contexts. To support the evaluation and training of data property matching techniques, we present a collection dataset consisting of product records from four different contexts. These datasets are the result of transforming two different existing datasets. In one of the datasets, some properties were filtered for being too noisy. The resulting processed dataset consists of json files with a listing of the product records and their properties, and a separate grouping of the properties that determines which ones match. It contains information about 2860 entities, with 4386 properties and 13350 pairwise matches.

## Specifications Table

**Table:** 

Subject	Applied Machine Learning
Specific subject area	Data integration and ontology matching
Type of data	JSON files
How data were acquired	Transformation of existing datasets containing product data
Data format	Raw Transformed
Parameters for data collection	None.
Description of data collection	Data from existing datasets containing information about products and their properties, as well as mapping of said properties to a reference ontology were processed to define groups of matching properties while grouping records by data source. Data properties mapped to a common reference property were considered a match
Data source location	http://di2kg.inf.uniroma3.it/2019/ http://webdatacommons.org/productcorpus/
Data accessibility	Repository name: LEAPME-datasets Data identification number: 10.5281/zenodo.5836484 Direct link to the dataset: https://github.com/dayala1/LEAPME-datasets
Related research article	D. Ayala, I. Hernández, D. Ruiz, E. Rahm, Leapme: Learning-based property matching with embeddings, Data & Knowledge Engineering (2021) 101,943. https://doi.org/10.1016/j.datak.2021.101943[1]

## Value of the Data

• The presented datasets offers a simple-to use collection real-world, multi-source product records with matched groups of data properties, which are related to the problem of schema matching.
• The datasets are useful for evaluating data-property matching techniques, specially those that match several data sources. They are also useful to users interested in training supervised classifiers for data property matching.
• The groups of matched properties can be used by researchers as examples of clusters, or property pairs by taking any pair of properties in the same group.
• Multi-source datasets match the real world data integration scenarios in which not two but an arbitrary number of data sources have to be integrated.
• Property groups include properties with the same meaning but completely different name, such as “resolution” and “pixels”.
• The availability of a large number of training examples is crucial for the possibility of eventually learning a context-independent universal property matching classifier.

## 1 Data Description

The LEAPME datasets [4] contain product records from 4 different real world e-commerce contexts: cameras, headphones, phones, and tvs. E-commerce data is ideal for property matching, since there is a large amount of data sources (the many existing commerce sites) with products of the same nature and therefore similar properties. Additionally, the extracted data from websites usually contains noise that makes it more realistic, as opposed to perfectly clean data that may make a trained model reliant on such conditions. The following files are provided:

• Folder “records” contains the product records. Each folder inside it corresponds to the data from one of the four contexts. Inside a context folder, each folder corresponds to a data source. Inside each data source, there are numbered json files corresponding to each record. Each record contains the value of data properties as key-value pairs. For example, file “records/cameras/cvp.com/10.json” corresponds to the 10th record from the “cvp.com” source for camera-related records. It contains the key-value properties related to that product, such as “coverage: Approx. 100%” and “weight: Approx. 217 g (including battery/batteries and memory card)”.
• Folder “mappings” contains the data property matches. Each json file in it corresponds to one of the four contexts. In each file, each key corresponds to a data properties group, which has a meaningful name for headphones, phones and tvs (e.g. “headphones_cup_type”) and a meaningless identifier for cameras (e.g. “TARGETATTRIBUTE#36”). Each group has an associated array of property names. Observe that two properties in the same group do not are necessarily equivalent, since other more complex relationships can also be present, such as m..n equivalencies. Therefore, a property of one source can match several properties of another source. For example, file “mappings/mappings-cameras.json” contains the following entry: “TARGETATTRIBUTE#27: [iso rating, iso max, iso min, …]”. Since “iso rating” and “iso min” are found in the same properties group, they are considered a match, even if they are not equivalent, since they are mapped to the same group. The same happens to “iso rating” and “iso max”. This happens because the ISO range of cameras is represented with a single property in one source (“iso rating”), and with two in a different source (“iso min” and “iso max”).
• File “property_instances.json” contains an easy-to-access collection of the instances of each data properties. First level keys correspond to each context. Second level keys correspond to each source in the context. Third level keys correspond to each data property in the source, and have an associated array with all the instances of said property.
• Folder “datasets as ontologies” contains the product records in owl format, following a similar structure to folder `mappings', but instead of having a folder for each data source, it contains an owl file.
• Folder “datasets original” contains the original datasets that were transformed to create the LEAPME datasets, as well as the scripts used to process them. Said datasets have been distributed for open use.

Table 1 shows statistics about our datasets. As can be observed, the cameras dataset is significantly larger than the rest when it comes to the number of sources, entities, properties, and instances. Because of the high number of entities per source, we set a limit of 100 entities per source in the cameras dataset, which makes it homogeneous. Apart from this homogeneity, several long tails can be observed in which one or a few sources of a context contain a much higher number of entities, properties or instances than the rest, since as expected some sources like ebay or amazon are larger than smaller stores, or have a more complete product information display.

**Table 1:** Datasets metadata. Each vertical bar in a plot represents a source within a dataset [1].

	Cameras	Headphones	Phones	TVs
# of Sources	24	6	12	8
Entities	2400	128	208	124
Properties	3245	172	554	415
Instances	65,615	1129	5195	2069
Positives	9199	412	2677	1062

				
Entities per source				

				
Instances per source				

				
Properties per source				

## 2 Experimental Design, Materials and Methods

The LEAPME datasets have been created by processing other existing products datasets, specifically from the DI2KG19 challenge cameras dataset [2] and the WDC Gold Standard for Product Matching and Product Feature Extraction dataset [3], containing information about headphones, phones, and tvs.

The original cameras dataset consisted of a xml file with a list of products, each with a set of properties, from which the “site” one denotes the source. A separate file mapped the property names to reference attributes. While a meaningful name was provided for the reference attributes, it was discarded due to it not being relevant for the property matching task. The xml file was transformed into the json files described in the former section, keeping the first 100 records for each data source. This work was done by script “datasets original/parse-cameras.py”, which iterates the products in the xml file, extracts the source site and the properties, and stores each record in a different file.

The original headphones, phones, and tv datasets consisted of a json file for each context describing the products of said context. The information about products was originally retrieved through web crawling techniques. Each product record includes some metadata and product properties divided according to the web element they come from: the product title, the product description, a web table, or a web list. We observed that the title and description properties were very noisy, so we only kept the list and table ones in the transformed files. This work was done by script “datasets/datasets original/parse-products.py”, which was ran for each of the three product categories (tvs, microphones, and phones). Each product in a category is iterated, extracting the source site, as well as the list and table attributes. Additionally, a mapping file is created in the same format as the one from the cameras dataset by using the “atts_map” field.

## Ethics Statements

Our work did not involve human subjects.

Our work did not involve animal experiments.

Our work did not involve data collected from social media platforms.

## CRediT authorship contribution statement

**Daniel Ayala:** Conceptualization, Methodology, Software, Data curation, Writing – original draft. **Inma Hernández:** Conceptualization, Writing – review & editing. **David Ruiz:** Writing – review & editing, Project administration, Funding acquisition. **Erhard Rahm:** Writing – review & editing, Supervision.

## Declaration of Competing Interest

The authors declare that they have no known competing financial interests or personal relationships which have or could be perceived to have influenced the work reported in this article.

## References

[1] Ayala D., Hernández I., Ruiz D., Rahm E. (2021). Leapme: learning-based property matching with embeddings. Data Knowl. Eng..

[2] Proceeding of the 1st International Workshop on challenges, Experiences from Data Integration to Knowledge Graphs, Di2kg Challenge, http://di2kg.inf.uniroma3.it/2019/, 2019. Accessed July 9, 2021. Reference to a book.

[3] Gold Standard for Feature Extraction. Web data commons, gold standard for product matching and product feature extraction, http://webdatacommons.org/productcorpus/, 2018. Accessed July 9, 2021.

[4] Ayala D. (2022). Dayala1/LEAPME-datasets: LEAPME datasets (dataset). Zenodo.

