# Corrigendum to Multi-source dataset of e-commerce products with attributes for property matching [Data in Brief 41 (2022) 107884]

**DOI:** 10.1016/j.dib.2022.108134

**Published:** 2022-04-02

**Authors:** Daniel Ayala, Inma Hernández, David Ruiz, Erhard Rahm

**Affiliations:** a Universidad de Sevilla, Seville, Spain; b Leipzig University, Leipzig, Germany

The authors regret having omitted a reference in the acknowledges section. It should read:

Our work was supported by the Spanish R&D&I Programme with grants PID2019–105471RB-I00, P18-RT-1060, and US-1380565 (Proyectos I+D+i FEDER Andalucía 2014-2020) US/JUNTA/FEDER, UE

The authors would like to apologise for any inconvenience caused.

